# Blastic plasmacytoid dendritic cell neoplasm secondary to acute myeloid leukemia with shared mutations in TET2 and DNMT3A: a case report and literature review

**DOI:** 10.3389/fimmu.2025.1656157

**Published:** 2025-12-10

**Authors:** Yao Sun, Yingjie Liu, Na Liu, Xiaohui Zhang, Wenrong Huang, Liangding Hu

**Affiliations:** 1 Senior Department of Hematology, Chinese People’s Liberation Army General Hospital, Beijing, China; 2 Beijing Garrison Haidian No. 38 Cadres Rest Home, Beijing, China

**Keywords:** BPDCN, AML, TET2, transdifferentiation, clonal hematopoiesis

## Abstract

Blastic plasmacytoid dendritic cell neoplasm (BPDCN) is a rare and aggressive hematologic malignancy. Recent studies have highlighted its occurrence in patients with a history of preceding myeloid neoplasms. This case report describes a patient who developed BPDCN secondary to acute myeloid leukemia (AML), with bone marrow involvement and clinical signs suggestive of CNS involvement. Genetic analysis revealed mutations in JAK2, DNMT3A, TET2, IKZF1, and MPL in BPDCN. Notably, TET2 and DNMT3A mutations were also present in the initial AML. A comprehensive review of existing literature identified 10 patients with BPDCN who had prior or concurrent hematologic malignancies, with detailed clonal data documented for each case. Among these, TET2 mutations emerged as a common feature, present in BPDCN and the associated hematologic malignancies in 9 of the 10 patients. Additionally, some of these patients exhibited early hematopoietic clones, diagnosed with lymphoma or secondary AML, with TET2 mutations consistently detected across all these conditions. These observations highlight the critical role of TET2 mutations in the development and progression of BPDCN and related hematologic neoplasms. However, the hierarchical structure of clonal evolution remains unclear, so this report also discusses the potential clonal relationships between different tumors.

## Introduction

Blastic plasmacytoid dendritic cell neoplasm (BPDCN) is a rare and aggressive hematological malignancy that primarily involves the skin, bone marrow (BM), and lymph nodes. With an estimated annual incidence of less than 1 per million, BPDCN predominantly affects elderly males and is associated with a dismal prognosis, with median survival ranging from 12 to 16 months despite intensive treatment regimens (1). The classification of BPDCN has evolved significantly over the years, and it is now categorized under dendritic cell and histiocytic tumors in the most recent 5th World Health Organization (WHO) Classification of Hematolymphoid Tumors (WHO-HEMA5). In WHO-HEMA5, the classification of histiocytic/dendritic cell neoplasms is placed after myeloid tumors because they originate from a common myeloid progenitor cell, forming the monocyte/histiocyte/dendritic cell lineage. This update reflects ongoing efforts to better understand the pathogenesis and cellular origins of this enigmatic neoplasm (2). Secondary BPDCN, which arises in the context of prior or concomitant hematologic malignancies (PCHM), has been reported in 10%–20% of cases (3). However, the relationship between clonal mutations in these two malignancies remains poorly understood. Recent studies have identified recurrent mutations in genes such as TET2, ASXL1, ZRSR2, and RAS, which are frequently observed in BPDCN (4, 5). Here, we report a case of BPDCN secondary to acute myeloid leukemia (AML) and provide a comprehensive review of cases in which the clonal relationship between BPDCN and associated hematologic neoplasms has been clearly established. This aims to deepen our understanding of this rare phenomenon and discuss the potential clonal relationships between different tumors.

## Case presentation

**Figure 1:**
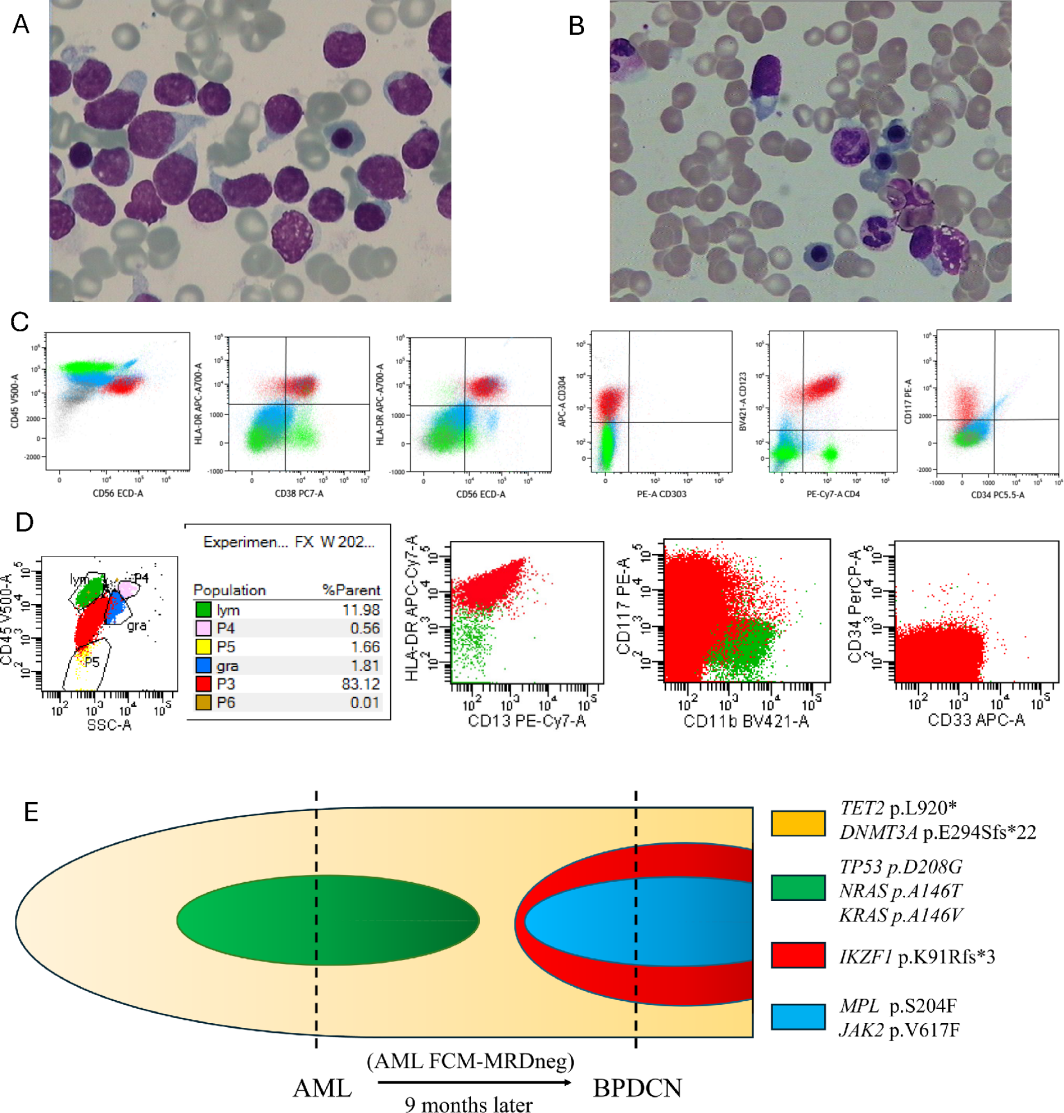
Morphological, immunophenotypic, and clonal features of AML and secondary BPDCN in the presented case. **(A)** Bone marrow morphology at AML diagnosis (Wright-Giemsa stain, ×100): Mononuclear cells with large size and irregular shape are observed, displaying a clustered distribution. The majority of the nuclei are round, with three atypical nucleoli. The chromatin is finely dispersed, and the cytoplasm is abundant. Pseudopodia formation is evident. **(B)** Bone marrow morphology at BPDCN progression (Wright-Giemsa stain, ×100): The tumor cells are of moderate size with irregular nuclei, exhibiting multiple nucleoli. The chromatin is finely granular, and the cytoplasm is abundant. Small vacuoles are seen near the cell membrane, demonstrating a “pearl necklace” appearance. **(C)** Flow cytometry immunophenotyping of AML blasts (red population): Expression of CD13, CD33, HLA-DR, and CD117. **(D)** Flow cytometry immunophenotyping of BPDCN cells (red population): Expression of HLA-DR, CD4, CD56, CD123, and CD304; partial CD33 expression. **(E)** Clonal evolution schematic: Shared TET2 and DNMT3A mutations (persisting from AML to BPDCN), alongside acquired mutations (*JAK2* p.V617E, *MPL* p.S204F, *IKZF1* p.K91Rfs3) specific to BPDCN progression. AML, Acute Myeloid Leukemia; BPDCN, Blastic Plasmacytoid Dendritic Cell Neoplasm; CNS, Central Nervous System; BM, Bone Marrow.

A 75-year-old male patient initially presented with fatigue and fever. Laboratory investigations revealed a significantly elevated white blood cell count of 39 × 10^9/L, a hemoglobin level of 102 g/L, and a platelet count of 62 × 10^9/L. Peripheral blood smear analysis revealed 86% blasts, suggestive of a hematologic malignancy. BM examination showed marked hypercellularity, with substantial suppression of both the granulocytic and erythroid lineages (**Figure 1A**). Blasts accounted for 97% of the marrow cellularity. Flow cytometry of the BM revealed that 83% of the cells expressed CD13, CD33, HLA-DR, CD117, CD38, CD6, CD7, and CD123, with partial expression of CD36 and CD371. These findings were consistent with the presence of malignant myeloid blasts (**Figure 1C**). Based on these investigations, a diagnosis of acute myeloid leukemia (AML) was established. Next-generation sequencing (NGS) revealed mutations in DNMT3A (41.06%), TET2 (43.32%), NRAS (1.07%), and TP53 (1.82%). Cytogenetic analysis showed a normal karyotype. The patient, classified as fit for intensive chemotherapy, was treated with the “7 + 3” regimen, which consists of 7 days of cytarabine and 3 days of idarubicin. Following this regimen, the patient achieved complete remission. Post-treatment flow cytometry analysis revealed no evidence of minimal residual disease. The patient and his family declined allogeneic hematopoietic stem cell transplantation. The patient subsequently underwent two additional cycles of intensive chemotherapy, followed by three courses of maintenance therapy with azacitidine and venetoclax.

Nine months after the initial diagnosis, the patient presented with new-onset vision impairment and unsteadiness in gait, which had developed over the past week. Upon admission, laboratory findings showed a white blood cell count of 1.29 × 10^9/L, hemoglobin of 115 g/L, and platelet count of 64 × 10^9/L. Peripheral blood smear again revealed 86% blasts, raising concern for AML relapse. The bone marrow morphology report revealed that unclassifiable cells accounted for 18% of nucleated cells (**Figure 1B**).​​ ​These cells exhibited medium size, with a round to irregular shape.​​ ​Their cytoplasm ranged from scanty to moderate in amount and displayed a characteristic basophilic (grayish-blue) hue, occasionally featuring a perinuclear halo.​​ ​The nuclear chromatin appeared coarse, with inconspicuous nucleoli.​​ ​Notably, some cells demonstrated pseudopodia and tailing projections.​​ ​Collectively, these morphological features raise strong suspicion of BPDCN.​ Immunophenotyping demonstrated the expression of markers consistent with BPDCN, including HLA-DR, CD4, CD7, CD38, CD56, CD117, CD123, and CD304, with partial positivity for CD33 (**Figure 1D**). These findings were suggestive of BPDCN rather than AML relapses. Notably, the patient did not exhibit any cutaneous manifestations, which are commonly observed in BPDCN, highlighting the atypical presentation in this case. Cytogenetic analysis remained normal. NGS revealed mutations in JAK2 (1.6%), DNMT3A (13.8%), TET2 (13%), IKZF1 (9.2%), and MPL (3.9%). Comparative genetic analysis revealed that the founding ​DNMT3A​ and ​TET2​ mutations persisted, while the clone had newly acquired mutations in ​JAK2, ​MPL, and ​IKZF1, illustrating a dynamic clonal evolution (**Figure 1E**). Although lumbar puncture was considered to evaluate potential CNS involvement, the procedure was declined by the patient’s family. Ultimately, the patient and family decided to forego further treatment, leading to the patient’s discharge home.

## Discussion

This case provides compelling evidence of a shared clonal origin between AML and secondary BPDCN, as demonstrated by the persistence of ancestral TET2 and DNMT3A mutations. Our literature review, in addition to the present case, identified 9 reported cases of BPDCN with preceding or concomitant hematologic malignancies and clearly documented clonal data, with involved sites at diagnosis encompassing the cutis, BM, and CNS (**Table 1**). Among previously reported cases of PCHM, 3 were simultaneous occurrences (2 MDS, 1 CMML), and 6 were previous events (4 CMML, 2 MDS). Of these previous events, 3 cases progressed to AML, with 1 case occurring after BPDCN. Among the 9 patients, except for one patient with MDS who did not show any detectable mutational clones, the remaining 8 patients all shared the TET2 mutation between BPDCN and PCHM. Notably, 3 of these patients had early clonal hematopoiesis data available, and one patient had previously been diagnosed with Anaplastic Lymphoma Kinase 1-negative Anaplastic Large Cell Lymphoma (ALK1-ALCL), with the shared TET2 clone present between all these conditions. As far as we know, this report presents the first case of *de novo* AML progressing to BPDCN, confirmed through shared mutations.

**Table 1:** Clonal Relationships Between BPDCN and PCHM.

Case (reference)	Clonal hematopoiesis mutations	Pre-BPDCN diagnosis 1 (disease & mutations)	Pre-BPDCN diagnosis 2 (disease & mutations)	BPDCN mutations	Shared mutations†	BPDCN involved sites
1 (21)	Not available	CMML: *TET2 p.G523Efs44, *SRSF2 p.P95L, *PHF6 p.Q251H, *PLCXD3 p.T282M, *TRMT61B p.T219C, STK3 p.N207Ifs3, *SLC25A10 p.R263C, *DIP2A p.R373W, *SARDH p.G641E, *PLP1 p.T116M, IVL p.P236_L245dup	–	*TET2 p.G523Efs44, *SRSF2 p.P95L, *PHF6 p.Q251H, *PLCXD3 p.T282M, *TRMT61B p.T219C, *STK3 p.N207Ifs3, *SLC25A10 p.R263C, *DIP2A p.R373W, *SARDH p.G641E, *PLP1 p.T116M, RB1 c.751C>T p.R251, CROCC p.T1532S, ERCC4 p.V870I, CHP2 p.R34Q	*TET2, *SRSF2, *PHF6, *PLCXD3, *TRMT61B, *STK3, *SLC25A10, *DIP2A, *SARDH, *PLP1	Cutis, BM
2 (22)	Not available	MDS: *SRSF2 p.P95H, *TET2 p.C1642fs53, *TET2 p.A1810fs	AML (from MDS): *ASXL1 p.E635fs15, CSF3R p.T618I, CREBBP p.Y1733fs, NRAS p.G12D, NRAS p.G12V, NRAS p.G12S*, *RUNX1 p.F1165, FLT3-ITD*	*SRSF2 p.P95H, *TET2 p.C1642fs53, *TET2 p.A1810fs	*TET2, *SRSF2	Cutis, BM
3 (20)	Not available	CMML (concomitant): *TET2 (specific mutation not provided)	–	*TET2 (same as CMML)	*TET2	Cutis, BM
4 (23)	Not available	CMML: *TET2 p.Y1244fsX1266, *TET2 p.Q810X, *SRSF2 P95H, JAK2 p.V617F	–	*TET2 p.Y1244fsX1266, *TET2 p.Q810X, *SRSF2 p.P95H	*TET2, *SRSF2	Cutis
5 (24)	*TET2 p.324-325IX fs, *TET2 p.P1915L, CUX1 p.R1214 fs, ATM p.M94V, NCOR1 sa, PHF6 p.K324	CMML: *TET2 p.324-325IX fs, *TET2 p.P1915L, CUX1 p.R1214 fs, ATM p.M94V, NCOR1 sa, PHF6 p.K324, RUNX1 p.S94N, TET2 p.L203X	AML (post-BPDCN): *TET2 p.324-325IX fs, *TET2 p.P1915L, CUX1 p.R1214 fs, ATM p.M94V, NCOR1 sa, PHF6 p.K324, RUNX1 p.S141L, CEBPA p.E76fs3, CEBPA p.Y285fs2, RUNX1 p.D198G,	*TET2 p.324-325IX fs, *TET2 p.P1915L, CUX1 p.R1214 fs, ATM p.M94V, NCOR1 sa, PHF6 p.K324, ASXL2 p.L1024fs18, ASXL2 p.P981A, ATM sd, CXCR4 p.I225fs7, CXCR4 p.W199R, MYC p.N383fs21, MYC p.R382fs8, RAD51C p.Q178*, STAT6 p.G600D,	*TET2	BM
6 (24)	AKAP6 p.G732S, *TET2 p.I771X	ALK-negative ALCL: *TET2 p.I771X, TP53 p.E286K, AKAP6 p.G732S, PRDM1 p.L664M, EP300 p.P1337L, JAK1 p.G1097A, NOTCH2 p.I2084insP, TET2 p.S5-61X del, TET2 p.73–1071 del	CMML: AKAP6 p.G732S, *TET2 p.I771X, TET2 p.Q150, *TET2 p.N1287S, SRSF2, SMARCA4, TP53 p.E285	BRCA1 sq, DNMT3A p.A145V, JAK2 p.D160Y, JAK3 p.Y633S, TP53 p.E285*, *TET2 p.N1287S, *TET2 p.I771X	*TET2	Cutis, BM
7 (25)	*TET2 p.Q1680X, *SF3B1 p.K700E	MDS (concomitant): *TET2 p.Q1680X, *SF3B1 p.K700E, TET2 p.R1167S, CUX1 p.Q318X, ZRSR2 p.L76X	–	*TET2 p.Q1680X, *SF3B1 p.K700E	*TET2, *SF3B1	Cutis
8 (26)	Not available	MDS: No mutations detected	–	*KRAS, NOTCH1, RUNX1 (specific mutations not provided)*	*None*	Cutis, BM, Eyes
9 (27)	CCUS: *ASXL1 p.Glu635Argfs15, *TET2 p.Asp143Ilefs2	MDS (concomitant): *ASXL1 p.Glu635Argfs15, *TET2 p.Asp143Ilefs2, *NRAS p.Gly12Asp	–	*ASXL1 p.Glu635Argfs15, *TET2 p.Asp143Ilefs2, *NRAS p.Gly12Asp	*ASXL1, *TET2, *NRAS	Cutis
Our case	Not available	AML: *DNMT3A p.E294Sfs*22, *TET2 p.L920* , NRAS p.A146T, KRAS p.A146V, TP53 p.D208G	–	*DNMT3A p.E294Sfs*22, *TET2 p.L920*, JAK2 p.V617E, MPL p.S204F, IKZF1 p.K91Rfs3	*TET2, *DNMT3A	BM, suspected CNS

† Shared Mutations: * indicates mutations shared between BPDCN and at least one prior or concomitant hematologic malignancy. Specific mutation sites are provided when available from original publications.

BPDCN, Blastic Plasmacytoid Dendritic Cell Neoplasm; PCHM, Prior or Concomitant Haematological Malignancies; CMML, Chronic Myelomonocytic Leukemia; MDS, Myelodysplastic Syndrome; AML, Acute Myeloid Leukemia; CH, Clonal Haematopoiesis; CCUS, Clonal Cytopenia of Undetermined Significance; ALK1, anaplastic lymphoma kinase 1; ALCL, Anaplastic Large Cell Lymphoma; CNS, Central Nervous System; BM, Bone Marrow; Cutis, skin involvement; VAF, Variant Allele Frequency.

TET2, which encodes a DNA dioxygenase essential for epigenetic regulation, is frequently mutated in clonal hematopoiesis (CH) (6). Griffin et al. (7) demonstrated that plasmacytoid dendritic cells harboring TET2 mutations exhibit resistance to UV-induced apoptosis, implicating this mutation in the pathogenesis of BPDCN. These findings suggest that TET2-driven epigenetic dysregulation may serve as a shared molecular foundation underlying divergent hematologic malignancies, warranting further mechanistic investigation. Although JAK2 and MPL mutations have not been previously linked to BPDCN, IKZF1 alterations have been reported in rare cases, suggesting a potential role in its pathogenesis that warrants further investigation (8).

Clonal hematopoiesis of indeterminate potential (CHIP) serves as a critical precursor to leukemogenesis, characterized by recurrent mutations—most frequently in DNMT3A, TET2, and ASXL1—that emerge in otherwise healthy individuals and significantly elevate the risk of AML and other myeloid neoplasms (9, 10). Foundational research confirms that preleukemic hematopoietic stem cells (HSCs) carrying DNMT3A and TET2 mutations can survive therapy and give rise to diverse malignant lineages (9, 10). In AML, large-scale genomic studies reveal substantial biological heterogeneity, with multiple subclones often coexisting at diagnosis and undergoing branched evolutionary trajectories at relapse (11, 12), frequently influenced by chemotherapy-induced selective pressures that reshape clonal architecture. In BPDCN, the high prevalence of marrow CH and the frequent sharing of TET2 mutations with associated myeloid malignancies support a model in which CH-derived ancestral clones undergo lineage-specific transformation driven by microenvironmental cues and secondary mutational events (5), consistent with the mechanism of UV-induced pDC leukemic transformation observed in the skin (7). In the present case, the disappearance of detectable TP53 at relapse despite persistent TET2/DNMT3A mutations suggests that relapse originated from a preleukemic ancestral clone rather than representing continued evolution of the initial TP53-mutant subclone, aligning with established models of AML clonal heterogeneity and evolution (11). Although TP53 mutations are generally associated with poor prognosis and often persist, their subclonal distribution and potential for eradication under therapeutic pressure have been documented (13).

Despite the common basis of TET2 mutations, the hierarchical relationships of clonal evolution in malignant clones during disease progression remain unknown. The mechanisms underlying the shared clonal origin of BPDCN and PCHM may involve two distinct pathways: divergent evolution from a common CH progenitor or transformation of BPDCN from an underlying myeloid neoplasm. Mature plasmacytoid dendritic cell proliferation (MPDCP) was officially mentioned only in WHO-5 and is associated with a myeloid neoplasm, frequently CMML, but also MDS or AML, particularly with monocytic differentiation (14–19). Another interesting phenomenon is that CMML cases have also been found to harbor plasmacytoid dendritic cell nodules (20). Therefore, BPDCN may potentially arise from these pDCs, which have already undergone early overproliferation or even clonal expansion, and further deteriorate due to a secondary hit. However, most institutions gave insufficient attention to flow cytometry analysis of pDCs in myeloid malignancies, resulting in limited exploration of the association between myeloid neoplasms and pDCs, and leaving the nature of this correlation still unclear. Therefore, when CMML patients or those with TET2 mutations present with clinical findings inconsistent with disease relapse, performing pDC analysis may help further elucidate the mechanisms underlying the development of secondary BPDCN. Future studies with larger cohorts are warranted to further elucidate the roles of TET2 and DNMT3A in the pathogenesis and clonal evolution of BPDCN.

We report a case of BPDCN secondary to *de novo* AML, characterized by shared TET2 and DNMT3A mutations. Our literature analysis underscores the pivotal role of TET2 mutations as a common genetic driver in the pathogenesis of BPDCN arising from myeloid neoplasms. Routine examination of pDCs in myeloid malignancies, particularly those harboring TET2 mutations, may be essential for elucidating the mechanisms underlying BPDCN development and its clonal evolution.

## Data Availability

The original contributions presented in the study are included in the article/Supplementary Material. Further inquiries can be directed to the corresponding authors.

## References

[1] Sasaki Y Murai S Shiozawa E Yamochi T Hattori N . Blastic plasmacytoid dendritic cell neoplasm in long-term complete remission after venetoclax monotherapy. Cureus. (2024) 16:e52446. doi: 10.7759/cureus.52446, PMID: 38371152 PMC10871153

[2] Khoury JD Solary E Abla O Akkari Y Alaggio R Apperley JF . The 5th edition of the world health organization classification of haematolymphoid tumours: myeloid and histiocytic/dendritic neoplasms. Leukemia. (2022) 36:1703–19. doi: 10.1038/s41375-022-01613-1, PMID: 35732831 PMC9252913

[3] Pagano L Valentini CG Pulsoni A Fisogni S Carluccio P Mannelli F . Blastic plasmacytoid dendritic cell neoplasm with leukemic presentation: an Italian multicenter study. Haematologica. (2013) 98:239–46. doi: 10.3324/haematol.2012.072645, PMID: 23065521 PMC3561431

[4] Pemmaraju N Kantarjian HM Khoury JD Loghavi S O’Brien S Cortes JE . Blastic plasmacytoid dendritic cell neoplasm (Bpdcn) commonly presents in the setting of prior or concomitant hematologic Malignancies (Pchm): patient characteristics and outcomes in the rapidly evolving modern targeted therapy era. Blood. (2019) 134:2723. doi: 10.1182/blood-2019-132185

[5] Khanlari M Yin CC Takahashi K Lachowiez C Tang G Loghavi S . Bone marrow clonal hematopoiesis is highly prevalent in blastic plasmacytoid dendritic cell neoplasm and frequently sharing a clonal origin in elderly patients. Leukemia. (2022) 36:1343–50. doi: 10.1038/s41375-022-01538-9, PMID: 35279700

[6] Hiwase D Hahn C Tran ENH Chhetri R Baranwal A Al-Kali A . Tp53 mutation in therapy-related myeloid neoplasm defines a distinct molecular subtype. Blood. (2023) 141:1087–91. doi: 10.1182/blood.2022018236, PMID: 36574363

[7] Griffin GK Booth CAG Togami K Chung SS Ssozi D Verga JA . Ultraviolet radiation shapes dendritic cell leukaemia transformation in the skin. Nature. (2023) 618:834–41. doi: 10.1038/s41586-023-06156-8, PMID: 37286599 PMC10284703

[8] Le Y Yu Q Zhu H Jiang Y Wang Z Li R . Protein structure prediction and bioinfamatic analysis of novel fusion gene ikzf1/pfas in bpdcn. Am Soc Hematol Washington DC. (2021). doi: 10.1182/blood-2021-148251

[9] Jaiswal S Fontanillas P Flannick J Manning A Grauman PV Mar BG . Age-related clonal hematopoiesis associated with adverse outcomes. New Engl J Med. (2014) 371:2488–98. doi: 10.1056/NEJMoa1408617, PMID: 25426837 PMC4306669

[10] Genovese G Kähler AK Handsaker RE Lindberg J Rose SA Bakhoum SF . Clonal hematopoiesis and blood-cancer risk inferred from blood DNA sequence. New Engl J Med. (2014) 371:2477–87. doi: 10.1056/NEJMoa1409405, PMID: 25426838 PMC4290021

[11] Ding L Ley TJ Larson DE Miller CA Koboldt DC Welch JS . Clonal evolution in relapsed acute myeloid leukaemia revealed by whole-genome sequencing. Nature. (2012) 481:506–10. doi: 10.1038/nature10738, PMID: 22237025 PMC3267864

[12] Papaemmanuil E Gerstung M Bullinger L Gaidzik VI Paschka P Roberts ND . Genomic classification and prognosis in acute myeloid leukemia. New Engl J Med. (2016) 374:2209–21. doi: 10.1056/NEJMoa1516192, PMID: 27276561 PMC4979995

[13] Döhner H Wei AH Appelbaum FR Craddock C DiNardo CD Dombret H . Diagnosis and management of aml in adults: 2022 recommendations from an international expert panel on behalf of the eln. Blood. (2022) 140:1345–77. doi: 10.1182/blood.2022016867, PMID: 35797463

[14] Zalmaï L Viailly PJ Biichle S Cheok M Soret L Angelot-Delettre F . Plasmacytoid dendritic cells proliferation associated with acute myeloid leukemia: phenotype profile and mutation landscape. Haematologica. (2021) 106:3056–66. doi: 10.3324/haematol.2020.253740, PMID: 33054115 PMC8634182

[15] Vitte F Fabiani B Bénet C Dalac S Balme B Delattre C . Specific skin lesions in chronic myelomonocytic leukemia: A spectrum of myelomonocytic and dendritic cell proliferations: A study of 42 cases. Am J Surg Pathol. (2012) 36:1302–16. doi: 10.1097/PAS.0b013e31825dd4de, PMID: 22895265

[16] Orazi A Chiu R O’Malley DP Czader M Allen SL An C . Chronic myelomonocytic leukemia: the role of bone marrow biopsy immunohistology. Modern Pathol: an Off J United States Can Acad Pathol Inc. (2006) 19:1536–45. doi: 10.1038/modpathol.3800707, PMID: 17041567

[17] Hamadeh F Awadallah A Meyerson HJ Beck RC . Flow cytometry identifies a spectrum of maturation in myeloid neoplasms having plasmacytoid dendritic cell differentiation. Cytometry Part B Clin Cytometry. (2020) 98:43–51. doi: 10.1002/cyto.b.21761, PMID: 30614203

[18] Huang Y Wang Y Chang Y Yuan X Hao L Shi H . Myeloid neoplasms with elevated plasmacytoid dendritic cell differentiation reflect the maturation process of dendritic cells. Cytometry Part A: J Int Soc Anal Cytol. (2020) 97:61–9. doi: 10.1002/cyto.a.23953, PMID: 31876105

[19] Xiao W Goldberg AD Famulare C Baik J Gao Q Tallman MS . Acute myeloid leukemia with plasmacytoid dendritic cell differentiation: predominantly secondary aml, enriched for runx1 mutations, frequent cross-lineage antigen expression and poor prognosis. Blood. (2018) 132:2789. doi: 10.1182/blood-2018-99-119081

[20] Hu Z Sun T . Blastic plasmacytoid dendritic cell neoplasm associated with chronic myelomonocytic leukemia. Blood. (2016) 128:1664. doi: 10.1182/blood-2016-06-723536, PMID: 28092882

[21] Patnaik MM Lasho T Howard M Finke C Ketterling RL Al-Kali A . Biallelic inactivation of the retinoblastoma gene results in transformation of chronic myelomonocytic leukemia to a blastic plasmacytoid dendritic cell neoplasm: shared clonal origins of two aggressive neoplasms. Blood Cancer J. (2018) 8:82. doi: 10.1038/s41408-018-0120-5, PMID: 30190511 PMC6127132

[22] Luskin MR Kim AS Patel SS Wright K LeBoeuf NR Lane AA . Evidence for separate transformation to acute myeloid leukemia and blastic plasmacytoid dendritic cell neoplasm from a shared ancestral hematopoietic clone. Leuk Lymphoma. (2020) 61:2258–61. doi: 10.1080/10428194.2020.1755856, PMID: 32366145

[23] Brunetti L Di Battista V Venanzi A Schiavoni G Martelli MP Ascani S . Blastic plasmacytoid dendritic cell neoplasm and chronic myelomonocytic leukemia: A shared clonal origin. Leukemia. (2017) 31:1238–40. doi: 10.1038/leu.2017.38, PMID: 28111467

[24] Denker S Künstner A Schwarting J Witte HM Bernard V Stölting S . Clonal evolution and blastic plasmacytoid dendritic cell neoplasm: Malignancies of divergent hematopoietic lineages emerging from a common founding clone. Leukemia. (2024) 38:1858–61. doi: 10.1038/s41375-024-02305-8, PMID: 38890446 PMC11286505

[25] Yamada T Hiramoto N Mori T Yamashita D Tai Y Yamamoto R . Coincidence of cutaneous blastic plasmacytoid dendritic cell neoplasm and myelodysplastic syndrome derived from clonal hematopoiesis. Blood Cancer J. (2023) 13:119. doi: 10.1038/s41408-023-00893-9, PMID: 37558659 PMC10412548

[26] Chamoun K Loghavi S Pemmaraju N Konopleva M Kroll M Nguyen-Cao M . Early detection of transformation to bpdcn in a patient with mds. Exp Hematol Oncol. (2018) 7:26. doi: 10.1186/s40164-018-0117-6, PMID: 30323983 PMC6174068

[27] Julin C Nielsen SL Grantzau TL Ahmad SA Cowland JB Bonde J . Localized blastic plasmacytoid dendritic cell neoplasm associated with progressive clonal hematopoiesis and myelodysplastic syndrome. APMIS: Acta Pathol Microbiol Immunol Scand. (2025) 133:e13486. doi: 10.1111/apm.13486, PMID: 39465574 PMC11649958

